# Online social connections and Internet use among people with intellectual disabilities in the United Kingdom during the COVID-19 pandemic

**DOI:** 10.1177/14614448221093762

**Published:** 2022-05-06

**Authors:** Sue Caton, Chris Hatton, Amanda Gillooly, Edward Oloidi, Libby Clarke, Jill Bradshaw, Samantha Flynn, Laurence Taggart, Peter Mulhall, Andrew Jahoda, Roseann Maguire, Anna Marriott, Stuart Todd, David Abbott, Stephen Beyer, Nick Gore, Pauline Heslop, Katrina Scior, Richard P Hastings

**Affiliations:** Manchester Metropolitan University, UK; University of Glasgow, UK; University of South Wales, UK; University of Warwick, UK; University of Kent, UK; University of Warwick, UK; University of Ulster, UK; University of Glasgow, UK; National Development Team for Inclusion, UK; University of South Wales, UK; University of Bristol, UK; University of Cardiff, UK; University of Kent, UK; University of Bristol, UK; University College London, UK; University of Warwick, UK; Monash University, Australia

**Keywords:** COVID-19, disability, intellectual disability, Internet, learning disability

## Abstract

Having a disability, in particular, an intellectual disability, is associated with Internet non-use. This article explores how people with intellectual disabilities used the Internet across the United Kingdom during the COVID-19 pandemic. In April to May 2021, 571 adults with intellectual disabilities were interviewed. Participants most commonly used the Internet for being with family and friends, social media or doing online activities with other people. People who lived with family were the most likely to use social media; people who lived with other people with intellectual disabilities were the least likely. People who self-reported as not lonely were more likely to use the Internet for online activities with others and play video games with others. Social connections were identified as the best thing about the Internet. Many participants chose not to identify a worst thing about Internet use, while others reported issues with technology, online harm and threats to well-being.

## Introduction

Despite Internet use being recognised as a necessity for participation in society (Eurostat, 2018), having a disability has been strongly related to Internet non-use (Helsper and Reisdorf, 2017). Disabled people have been shown to have less access to devices (Johansson et al., 2021; Pew Research Center, 2021a) as well as using the Internet less to pay bills, using the Internet less for online shopping and feeling less included in the digital society than the general population (Johansson et al., 2021). Disabled people are not a homogeneous group though and there are several digital divides facing disabled people with differences between disability groups in access to devices, use of the Internet and in perceived difficulties in use of the Internet (Johansson et al., 2021). Fewer people with language disabilities and people with intellectual disabilities experience access to devices and are also the most likely to report difficulties in using the Internet (Johansson et al., 2021). People with intellectual disabilities are often socially isolated and commonly have less education and experience greater unemployment than non-disabled people; these inequalities are known to be associated with being digitally excluded (Helsper and Reisdorf, 2017; Hoppestad, 2013).

Barriers that prevent people with intellectual disabilities from full digital participation include individual differences as well as wider societal attitudes and technological accessibility (Chadwick et al., 2019). Specific barriers include the restrictive financial barriers associated with devices and data (Alfredsson Ågren et al., 2020; Chadwick et al., 2013), the often challenging technical requirements of getting online (Lussier-Desrochers et al., 2017), accessibility of websites (Shpigelman and Gill, 2014; Williams and Hanson-Baldauf, 2010), lower levels of literacy (Caton and Chapman, 2016), safeguarding restrictions placed on people with intellectual disabilities by the people who support them (Barlott et al., 2019; Heitplatz et al., 2021; Löfgren-Mårtenson, 2008) and limited knowledge of people who support people with intellectual disabilities to be able to assist with devices or online platforms (Alfredsson Ågren et al., 2020; Caton and Landman, 2021). There are also more nuanced barriers such as interpreting the online behaviours of others (Caton and Chapman, 2016; Lough and Fisher, 2016) and comprehension of social codes and conventions when using the Internet (Lussier-Desrochers et al., 2017). These barriers manifest both in access to Internet-enabled devices (Alfredsson Ågren et al., 2019; Glumbić et al., 2021), and in the way, people with intellectual disabilities use the Internet (Alfredsson Ågren et al., 2019).

Despite these difficulties, more recent small-scale studies have suggested that given the opportunity and technology, people with intellectual disabilities, especially younger people (Alfredsson Ågren et al., 2020), are gradually becoming more digitally included with increased ownership of Internet-enabled smartphones (Chiner et al., 2017). This includes a growing understanding of the benefits of Internet use being acknowledged by people who support people with intellectual disabilities (Caton and Landman, 2021). Active Internet use in people with intellectual disabilities has been shown to often be a positive experience enabling the maintenance of existing social connections (Barlott et al., 2019; Raghavendra et al., 2018; Shpigelman, 2018; Shpigelman and Gill, 2014). The use of mobile technology, in particular, frequent and self-determined use, has been positively associated with social inclusion with family, friends and work or volunteering (Martin et al., 2021).

There is no reliable information on the number of adults with intellectual disabilities in the United Kingdom (Hatton et al., 2016), although up to three quarters of adults with intellectual disabilities are thought not to be identified by any health or social care service. Statistics concerning uptake of services and living situations are collected separately in England, Scotland, Wales and Northern Ireland using methods that are inconsistent with each other. Overall, most adults with intellectual disabilities known to health or social care services are living with family members, with rising numbers of adults living in supported living services and decreasing numbers of adults living in residential care. Rates of paid employment among adults with intellectual known to social care services are extremely low (less than 10%), and largely consist of low hours, low-wage work (NHS Digital, 2022). There are long-term trends towards reductions in day centre places, with support during the day more often in the form of personal assistants, although investment in social care has not kept pace with those who need such support (Forrester-Jones et al., 2021).

The worldwide COVID-19 pandemic and associated national and regional lockdown policies have placed restrictions on most aspects of people’s lives but underlined the importance of Internet use. For people with intellectual disabilities, the closure of services, community activities and associated routines led to a disruption to informal and formal social support and people with intellectual disabilities found themselves isolated from the people in their lives who supported them socially and emotionally (Flynn et al., 2021; Lake et al., 2021). The impact of the disruption of continuity of support led to the rapid uptake of other ways to maintain social connections with people such as an increased use of the telephone and connecting with friends online (Chadwick et al., 2022; Lake et al., 2021) and research has suggested that digital skills were needed to navigate the disruption (Spencer et al., 2021). In recognition of this need, local and national initiatives have facilitated more people with intellectual disabilities to get digitally connected (e.g. In England, the government-funded Digital Lifeline initiative distributed 5500 devices to people with intellectual disabilities; Good Things Foundation, 2021), and the development of resources to increase digital knowledge and skills (Seale, 2021).

In this study, we explore how people in the United Kingdom with mild to moderate intellectual disabilities used the Internet during the COVID-19 pandemic specifically addressing the range of online activities, and age, gender and living circumstances factors associated with digital use. In addition, we explore the experiences of Internet use for people with intellectual disabilities during the pandemic by examining what participants identified as positive and negative aspects of Internet use and associations between digital use and social connectedness.

The study draws on both quantitative and qualitative data collected during the second wave of the Coronavirus and People with Learning Disabilities Study (April to May 2021) just over a year after the first UK national lockdown. The wider study involved two cohorts of participants. Cohort 1 were adults with mild to moderate intellectual disabilities who were interviewed by a researcher, Cohort 2 were family carers and support workers of adults with severe/profound intellectual disabilities who were not able to take part in an interview themselves, who participated through an online survey. Slightly different questions were asked of each Cohort with more questions about Internet use asked of Cohort 1. Therefore, this study includes data from Cohort 1 only. *Researchers directly interviewed people* with intellectual disabilities about their experiences during the pandemic, including their experiences of Internet use during periods of national and regional restrictions. *Data collected were mainly quantitative with responses entered directly into Qualtrics*^™^ *during the interviews by the researchers. Four open-ended questions relating to experiences of Internet use were also included providing more detailed qualitative data.*

## Method

### Participants

Adults with intellectual disabilities (*N* = 571) were interviewed remotely using Zoom, telephone, WhatsApp, Teams or FaceTime (according to the preference of the participant) during April and May 2021. Most were aged 16–44 years (70.4%) with the remainder older, and just over half were men (51.0%). Over a third were living with their family (39.4%), just under a third (32.5%) were living alone or with a partner, and over a quarter (28%) were living with other people with intellectual disabilities (this included supported living and residential care). Participants lived in Wales (30.8%), England (27.1%), Scotland (23.9%) and Northern Ireland (18.1%), and 95.7% of participants identified as white (Welsh, English, Scottish, Northern Irish, British, Irish, Gypsy or Irish Traveller).

### Measures

Table 1 shows the set of questions used for the analyses in this article, including how they were coded for the purposes of analysis. Responses reported by small numbers of participants were collapsed into broader categories or excluded from the specific analyses involving the relevant variable, but these participants were included in all other analyses (e.g. people identifying as a gender other than male or female were excluded from analyses concerning gender differences, but were included in all other analyses). Where response options were collapsed into a smaller number of categories for analysis, this was done on the basis of creating meaningful categories for analysis with sufficient numbers in each category. For the analyses included here questions related to demographic factors, Internet usage, and social connection and loneliness. Loneliness was measured using an adapted version of a question (‘in the last 4 weeks, how often did you feel lonely with no one to talk to?’) from Great Britain’s Office for National Statistics Opinions and Lifestyle Survey (ONS, 2020a).

**Table 1.** Measures and participant data used for analyses reported here.

Measure and coding	Cohort 1 (interviews with adults with intellectual disabilities, %)
Demographic variables	
Age	
16–34	*n* = 262 (45.9)
35–54	*n* = 231 (40.5
55+	*n* = 78 (13.7)
Gender	
Male	*n* = 296 (52.0)
Female	*n* = 273 (48.0)
Living situation	
Living with family	*n* = 228 (39.4)
Living alone/with partner	*n* = 189 (32.6)
Living with other people with intellectual disabilities	*n* = 162 (28.0)
Internet usage	
Internet access at home	
Yes	*n* = 529 (89.8)
Internet at home but do not use it and no internet at home	*n* = 60 (10.2)
Online activities organised by self-advocacy group in last 4 weeks	
Yes	*n* = 239 (40.8)
No	*n* = 347 (59.2)
What do you use the Internet for at the moment	
Being with friends/family online	*n* = 391 (66.4)
Social media	*n* = 357 (60.6)
Doing online activities with other people	*n* = 345 (58.6)
Streaming TV and films	*n* = 318 (54.0)
Other activities on your own	*n* = 299 (50.8)
Shopping	*n* = 215 (36.5)
Playing video games with other people online	*n* = 114 (19.4)
For paid or voluntary work	*n* = 112 (19.0)
Time spent each day using the Internet for paid or voluntary work (of those using the Internet for work)	
Not every day	*n* = 51 (45.9)
Up to 4 hours a day	*n* = 37 (33.3)
More than 4 hours a day	*n* = 23 (20.7)
Time spent each day using the Internet for other reasons than paid or voluntary work (of those using the Internet for other reasons)	
Not every day	*n* = 57 (11.1)
Up to 4 hours a day	*n* = 264 (51.4)
More than 4 hours a day	*n* = 193 (37.5)
Social connectedness	
Felt lonely with no one to talk to in the last 4 weeks	
Never or hardly ever	*n* = 292 (49.8)
Some of the time and often or always	*n* = 294 (50.2)
Staying in touch with family and friends in the last 4 weeks	
Yes, as much as I want	*n* = 471 (80.1)
Yes, but not sometimes and No	*n* = 117 (19.9)

Participants were also asked open-ended questions which included ‘During the coronavirus situation, what have been the best things about having the internet?’ and ‘During the coronavirus situation, what have been the worst things about having the internet?’ Interviewers were instructed to record responses verbatim where possible. We report on analyses of participants’ responses to these questions and anonymised quotes are used to illustrate some points. In addition, we asked, ‘Do you want your life to go back to how it was before coronavirus, or would you like some things to be different to how they were before?’ and ‘Since the early days of the pandemic in 2020, how keen have you been to take part in online activities?’ and we report on analyses of participants’ responses to these questions.

### Procedure

The selection and wording of questions for interviews were finalised through extensive consultation with groups of people with intellectual disabilities across the four UK countries to maximise relevance and accessibility. Following initial consultation on the selection of questions, the wording of draft questions was further discussed with members of the study’s collaborating organisations to ensure accessibility, and adjustments were made where necessary. Recruitment of people into the study was facilitated through multiple methods, including through collaborating organisations in each UK country, social media and wider networks of intellectual disability and family organisations across England, Northern Ireland, Scotland and Wales. Potential participants could express interest in the study through telephone, e-mail, social media, or through the research project website. Contact details of people who had indicated an interest in taking part in interviews were sent to research teams in each country, who contacted each person to talk through the project and send them the easy read participant information sheet. If people were still interested in taking part, at least 24 hours later the interviewer arranged to go through the consent process and, if the person consented, conduct the interview.

Data were collected in April and May 2021 by trained research interviewers who directly interviewed adults with intellectual disabilities through Zoom, telephone, Microsoft Teams, WhatsApp video call, Messenger video call or FaceTime, depending on the interviewee’s preference. All interviewees had the capacity to take part in the interviews and gave their consent to do so before the interview was conducted. Data were entered directly into Qualtrics^™^ by the interviewers. One person preferred to self-complete an online version of the survey, and this was made available to them at their request. Participants were also able to have a supporter of their choice (e.g. family member, support staff) present at the interview. In all cases, flexibility was paramount to ensure that people with intellectual disabilities were able to participate in their preferred way. Interviews took typically 45 minutes to complete and were usually completed in one sitting. Short breaks were offered during interviews when needed. All interviewers had experience of research interviewing and were trained through online training sessions within each country, with additional training sessions across all UK interviewers and regular supervision for interviewers.

### Analysis

For quantitative analyses, the dataset was analysed using SPSS 26. For categorical variables, potential associations involving Internet usage and other factors were analysed using Fisher’s exact test (for 2 × 2 tables) or Chi-square (for 3 × 2 and 3 × 3 tables) for all comparisons. All tests were two-sided. As the research was exploratory, the statistical significance level was set at *p* < .05 with no adjustments for multiple comparisons. To assist in interpretations of effect size, Relative Risk (RR) calculations were also conducted. As these are conducted for 2 × 2 tables, where a variable had more than two categories (e.g. age band), a series of binary variables was constructed (e.g. age 18–34 vs all other ages; age 35–54 vs all other ages; age 55+ vs all other ages) and a set of RR calculations was conducted using these binary variables. The three age bands used in the analysis were based on patterns of frequency of Internet use by age group of adults in Great Britain (ONS, 2020b).

For qualitative analyses, qualitative content analysis (an interpretive form of content analysis; Hsieh and Shannon, 2005) was used to develop themes in the data. To establish reliability, a pair of authors independently reviewed the responses to each question to develop an initial thematic coding framework for each question. Frameworks were then compared until an agreement on a final list of codes was reached. The pair of authors independently coded 20% of the data (O’Connor and Joffe, 2020). Some participants’ responses contained more than one concept, so each new concept was recorded separately but each concept was only coded to one code. We ensured there was a Kappa coefficient of at least .61 between raters for each code, which is considered to be substantial agreement (Landis and Koch, 1977), by discussing any differences and making minor adjustments to the coding approach. Once this level of correspondence was established, and any disagreements were resolved with second raters, the first author adjusted the coding approach and continued to code the full data sets.

### Ethical approval

Research ethics approval was sought and obtained from the Faculty of Health, Psychology and Social Care Faculty Research Ethics Committee at Manchester Metropolitan University. Informed audio-recorded consent was obtained from each participant before the interview began.

## Results

Table 1 presents descriptive data on Internet usage among adults with intellectual disabilities across the United Kingdom. The vast majority of people (89.8%) used the Internet at home. People most commonly used the Internet for being with family and friends online (66.4%), social media such as Facebook or Instagram (60.6%), doing online activities with other people (58.6%), streaming TV and films (54.0%) and doing other online activities on their own (50.8%). A substantial minority of people (40.8%) had been involved in online activities organised by a self-advocacy group in the 4 weeks before being interviewed. A minority of people were using the Internet for paid or voluntary work purposes (19.0%).

Those using the Internet for paid or voluntary work purposes (*N* = 112) were most commonly not using the Internet for this purpose every day (45.9%), with 33.3% using the Internet up to 4 hours a day for this purpose and 20.7% using the Internet more than 4 hours a day for this purpose. The vast majority of the much larger number of people using the Internet for other purposes were using the Internet daily (88.9%), with 37.5% of people using the Internet for more than 4 hours a day.

Table 2 shows potential associations between indicators of Internet usage and the demographic variables of age (in three age bands), gender, and living situation. As Table 2 shows, the youngest (16–34 years) and oldest (55+ years) age groups were associated with a wide range of indicators of Internet usage. People aged 16–34 were three times more likely to use the Internet at home, more likely to use the Internet for the purposes of being with family and friends online (60% more likely), streaming TV and films (70% more likely), playing video games with other people online (58% more likely), social media (twice as likely), and online shopping (39% more likely), and 45% more likely to spend 4 hours or more on the Internet for reasons other than paid/voluntary work. People aged 55+ were 72% less likely to use the Internet at home, less likely to use the Internet for the purposes of being with family and friends online (62% less likely), streaming TV and films (63% less likely), playing video games with other people online (78% less likely), social media (77% less likely), and online shopping (74% less likely), and 42% more likely to not use the Internet every day for reasons other than paid/voluntary work. People in the 35–54 age group were also less likely to use the Internet for the purposes of streaming TV and films (22% less likely), playing video games with other people online (27% less likely) and social media (21% less likely), and 25% less likely to spend 4 hours or more on the Internet for reasons other than paid/voluntary work.

**Table 2.** Associations between Internet usage and demographic variables.

	Demographic variables^a^		
	Age (16–34 vs 35–54 vs 55+)	Gender (man vs woman)	Living situation (living alone/with partner vs living with family vs living with other people with intellectual disabilities)
Internet usage			
Internet access at home	96.2% vs 88.7% vs 70.5% **Chi-square** **=** **43.11; *df*** **=** **2; *p*** **<** **.001** **RR (16–34 vs all other ages)** **=** **2.90 (1.64–5.14)** **RR (55+ vs all other ages)** **=** **0.28 (0.18–0.41)**	89.5% vs 89.7% Fisher’s *p* = 1.000 RR = 0.998 (0.94–1.06)	85.2% vs 93.4% vs 89.5% **Chi-square** **=** **7.55; *df*** **=** **2; *p*** **=** **0.023** **RR (living alone vs all other living situations)** **=** **0.93 (0.87–0.99)** **RR (family vs all other living situations)** **=** **1.07 (1.02–1.13)**
Online activities organised by self-advocacy group in last 4 weeks	42.7% vs 38.6% vs 35.9% Chi-square = 1.55; *df* = 2; *p* = .460	42.7% vs 38.4% Fisher’s *p* = 0.305 RR = 1.11 (0.91–1.36)	36.9% vs 44.7% vs 39.8% Chi-square = 2.72; *df* = 2; *p* = .257
What do you use the Internet for at the moment			
Being with friends/family online	75.6% vs 62.8% vs 42.3% **Chi-square** **=** **31.21; *df*** **=** **2; *p*** **<** **.001** **RR (16–34 vs all other ages)** **=** **1.60 (1.28–2.00)** **RR (55+ vs all other ages)** **=** **0.38 (0.25–0.58)**	67.2% vs 64.8% Fisher’s *p* = 0.595 RR = 1.04 (0.92–1.17)	63.5% vs 70.2% vs 64.2% Chi-square = 2.52; *df* = 2; *p* = .284
Doing online activities with others	59.9% vs 58.4% vs 50.0% Chi-square = 2.47; *df* = 2; *p* = .292	58.8% vs 57.9% Fisher’s *p* = 0.865 RR = 1.02 (0.88–1.17)	55.0% vs 57.9% vs 63.0% Chi-square = 2.30; *df* = 2; *p* = .317
For paid or voluntary work	18.7% vs 22.1% vs 12.8% Chi-square = 3.31; *df* = 2; *p* = 0.191	19.3% vs 19.0% Fisher’s *p* = 1.000 RR = 1.01 (0.72–1.42)	23.3% vs 18.9% vs 15.4% Chi-square = 3.50; *df* = 2; *p* = .174
Streaming TV and films	66.0% vs 47.2% vs 29.5% **Chi-square** **=** **38.31; *df*** **=** **2; *p*** **<** **0.001** **RR (16–34 vs all other ages)** **=** **1.70 (1.39–2.06)** **RR (35–54 vs all other ages)** **=** **0.78 (0.64–0.95)** **RR (55+ vs all other ages)** **=** **0.37 (0.23–0.58)**	56.8% vs 49.1% Fisher’s *p* = 0.077 RR = 1.16 (0.99–1.35)	48.1% vs 57.5% vs 54.9% Chi-square = 3.74; *df* = 2; *p* = .154
Playing video games with others	27.9% vs 15.2% vs 5.1% **Chi-square** **=** **24.60; *df*** **=** **2; *p*** **<** **.001** **RR (16–34 vs all other ages)** **=** **1.58 (1.33–1.88)** **RR (35–54 vs all other ages)–0.73 (0.55–0.98)** **RR (55+ vs all other ages)** **=** **0.22 (0.08–0.59)**	26.0% vs 11.7% **Fisher’s *p*** **<** **0.001** **RR** **=** **2.22 (1.52–3.24)**	14.8% vs 23.7% vs 17.9% Chi-square = 5.48; *df* = 2; *p* = .065 **RR (family vs all other living situations)** **=** **1.46 (1.05–2.03)**
Social media	75.6% vs 54.5% vs 25.6% **Chi-square** **=** **67.83; *df*** **=** **2; *p*** **<** **.001** **RR (16–34 vs all other ages)** **=** **2.04 (1.63–2.56)** **RR (35–54 vs all other ages)** **=** **0.79 (0.65–0.97)** **RR (55+ vs all other age groups)** **=** **0.23 (0.14–0.37)**	56.8% vs 63.4% Fisher’s *p* = 0.123 RR = 0.90 (0.78–1.02)	61.9% vs 68.9% vs 46.9% **Chi-square** **=** **19.33; *df*** **=** **2; *p*** **<** **.001** **RR (family vs all other living situations)** **=** **1.25 (1.10–1.42)** **RR (live with other people with intellectual disabilities vs all other living situations)** **=** **0.71 (0.60–0.85)**
Shopping	44.3% vs 35.5% vs 12.8% **Chi-square** **=** **25.82; *df*** **=** **2; *p*** **<** **.001** **RR (16–34 vs all other ages)** **=** **1.39 (1.17–1.65)** **RR (55+ vs all other ages)** **=** **0.26 (0.14–0.49)**	29.4% vs 42.9% **Fisher’s *p*** **<** **0.001** **RR** **=** **0.69 (0.55–0.86)**	41.3% vs 35.1% vs 32.7% Chi-square = 3.05; *df* = 2; *p* = .217
Other activities on your own	51.9% vs 49.8% vs 43.6% Chi-square = 1.67; *df* = 2; *p* = .435	55.1% vs 45.4% **Fisher’s *p*** **=** **0.024** **RR** **=** **1.21 (1.03–1.43)**	49.2% vs 54.4% vs 46.3% Chi-square = 2.65; *df* = 2; *p* = .266
Time spent each day using the Internet for paid/voluntary work			
Not every day	45.8% vs 50.0% vs 20.0%	48.1% vs 43.1%	43.2% vs 41.5% vs 56.5%
Up to 4 hours a day	29.2% vs 33.3% vs 60.0%	37.0% vs 29.4%	34.1% vs 34.1% vs 30.4%
More than 4 hours a day	25.0% vs 16.7% vs 20.0%	14.8% vs 27.5%	22.7% vs 24.4% vs 13.0%
	Chi-square = 4.78; *df* = 4; *p* = .310	Chi-square = 2.60; *df* = 2; *p* = .272	Chi-square = 1.85; *df* = 4; *p* = .763
Time spent each day using the Internet for other reasons than paid/voluntary work			
Not every day	4.1% vs 15.5% vs 30.8%	13.4% vs 8.9%	11.4% vs 6.8% vs 17.1%
Up to 4 hours a day	49.6% vs 53.5% vs 50.0%	50.2% vs 53.8%	46.2% vs 55.3% vs 50.0%
More than 4 hours a day	46.3% vs 31.0% vs 19.2% **Chi-square** **=** **43.94; *df*** **=** **4; *p*** **<** **0.001** **RR (not every day; people aged 16–34)** **=** **0.33 (0.19–0.58)** **RR (not every day; people aged 35–44)** **=** **1.42 (1.09–1.85)** **RR (not every day; people aged 55+)** **=** **3.44 (2.04–5.79)** **RR (more than 4 hours per day; people aged 16–34)** **=** **1.45 (1.22–1.72)** **RR (more than 4 hours per day; people aged 35–54)** **=** **0.75 (0.59–0.96)** **RR (more than 4 hours per day; people aged 55+)** **=** **0.40 (0.21–0.78)**	36.4% vs 37.3% Chi-square = 2.58; *df* = 2; *p* = .275	42.4% vs 37.9% vs 32.9% **Chi-square** **=** **11.34; *df*** **=** **4; *p*** **=** **.023** **RR (not every day; family vs all other living situations)** **=** **0.48 (0.27–0.86)** **RR (not every day; live with other people with intellectual disabilities vs all other living situations)** **=** **1.95** **(1.19–3.19)**

a For 3 × 2 and 3 × 3 tables, Relative Risk (RR) statistics are only reported when 95% CIs do not pass through 1.

There were few gender differences in Internet usage: men were more likely to use the Internet to play video games with other people online (more than twice as likely; Fisher’s *p* < 0.001) and to use the Internet for other purposes on their own (21% more likely; Fisher’s *p* < 0.05), and women were more likely to use the Internet for shopping (45% more likely; Fisher’s *p* < 0.001).

Regarding living situation, people living with their family were 7% more likely to have access to the Internet at home (*p* < .05) than people in other living situations. People who lived with their family were most likely (25% more likely) and people who lived with other people with intellectual disabilities were least likely (29% less likely) to use the Internet for social media purposes (*p* < 0.001). People living with their family (52% less likely) and people living with other people with intellectual disabilities (almost twice as likely) were more or less likely to be using the Internet less than daily for non-work purposes (*p* < .05).

In terms of social connectedness, almost half the adults we interviewed reported that in the last 4 weeks they never or hardly ever felt lonely (49.8%), with a further 35.5% of people feeling lonely some of the time and 14.7% of people feeling lonely often or always. A majority of people (80.0%) reported that they had been staying in touch with family and friends as much as they wanted in the last 4 weeks. Table 3 shows potential associations between indicators of Internet usage and two indicators of social connectedness: loneliness, and whether people were in touch with family and friends as much as they wanted. As Table 3 shows, people who reported themselves to be never or hardly ever lonely were more likely to do online activities with others (16% more likely) and play video games with others online (49% more likely; both *p* < .05) than people who reported themselves to be sometimes, often or always lonely. There were no associations between any indicator of Internet usage and whether people were in touch with family and friends as much as they wanted to be.

**Table 3.** Associations between Internet usage and social connectedness.

	Feeling lonely (never/hardly ever vs some/often/all the time)	Staying in touch (as much as I want vs no/sometimes no)
Internet access at home	91.4% vs 88.4% Fisher’s *p* = 0.272 RR = 1.03 (0.98–1.09)	89.6% vs 91.5% Fisher’s *p* = 0.611 RR = 0.98 (0.92–1.04)
Online activities organised by self-advocacy group in last 4 weeks	39.7% vs 42.3% Fisher’s *p* = 0.556 RR = 0.94 (0.77–1.14)	41.7% vs 37.6% Fisher’s *p* = 0.462 RR = 1.11 (0.86–1.43)
What do you use the Internet for at the moment		
Being with friends/family online	67.8% vs 65.6% Fisher’s *p* = 0.600 RR = 1.03 (0.92–1.16)	67.1% vs 64.1% Fisher’s *p* = 0.584 RR = 1.05 (0.90–1.22)
Doing online activities with others	63.0% vs 54.4% **Fisher’s *p*** **=** **0.036** **RR** **=** **1.16 (1.01–1.33)**	58.6% vs 59.0% Fisher’s *p* = 1.000 RR = 0.99 (0.84–1.18)
For paid or voluntary work	16.4% vs 21.8% Fisher’s *p* = 0.115 RR = 0.76 (0.54–1.06)	19.7% vs 16.2% Fisher’s *p* = 0.432 RR = 1.22 (0.78–1.91)
Streaming TV and films	56.2% vs 52.4% Fisher’s *p* = 0.363 RR = 1.07 (0.92–1.24)	55.6% vs 47.9% Fisher’s *p* = 0.147 RR = 1.16 (0.95–1.43)
Playing video games with others	23.3% vs 15.6% **Fisher’s *p*** **=** **0.022** **RR** **=** **1.49 (1.06–2.09)**	18.9% vs 21.4% Fisher’s *p* = 0.601 RR = 0.88 (0.60–1.31)
Social media	60.6% vs 60.5% Fisher’s *p* = 1.000 RR = 1.00 (0.88–1.14)	59.4% vs 65.8% Fisher’s *p* = 0.245 RR = 0.90 (0.78–1.05)
Shopping	35.3% vs 38.1% Fisher’s *p* = 0.494 RR = 0.93 (0.75–1.15)	36.3% vs 37.6% Fisher’s *p* = 0.830 RR = 0.97 (0.74–1.26)
Other activities on your own	51.7% vs 50.3% Fisher’s *p* = 0.742 RR = 1.03 (0.88–1.20)	50.3% vs 53.0% Fisher’s *p* = 0.608 RR = 0.95 (0.78–1.15)
Time spent each day using the Internet for paid/voluntary work		
Not every day	45.8% vs 45.0%	44.6% vs 50.0%
Up to 4 hours a day	29.2% vs 36.7%	33.7% vs 31.3%
More than 4 hours a day	25.0% vs 18.3%	21.7% vs 18.8%
	Chi-square = 1.01; *df* = 2; *p* = .603	Chi-square = 0.17; *df* = 2; *p* = .918
Time spent each day using the Internet for other reasons than paid/voluntary work		
Not every day	11.8% vs 10.4%	10.2% vs 14.4%
Up to 4 hours a day	51.1% vs 51.4%	52.7% vs 46.2%
More than 4 hours a day	37.0% vs 38.2%	37.1% vs 39.4%
	Chi-square = 0.30; *df* = 2; *p* = .859	Chi-square = 2.12; *df* = 2; *p* = 0.347

Table 4 shows the broad range of activities that were identified by participants as the best things about having the Internet during the pandemic. The number and percentage of participants mentioning each activity are shown in Table 4. No single activity was mentioned by more than 52% of participants, so due to the range of activities, codes were categorised into five themes. In order of number of mentions by participants, these were social connections; entertainment; life, work and education; information seeking; and technology.

**Table 4.** Coded best things about the Internet during the coronavirus pandemic.

	Number (and %) of participants mentioning theme (total sample = 538)	Number (and %) of participants mentioning item (total sample = 538)
Social connections	**419 (78)**	
Live online activities or groups		84 (16)
Keeping in touch with friends, family and work colleagues		280 (52)
Meeting new people		26 (5)
Social media		29 (5)
Entertainment	**165 (31)**	
Watching TV or videos (e.g. YouTube)		80 (15)
Gaming		34 (6)
Individual activities (e.g. puzzles, music)		17 (3)
Life, work and education	**124 (23)**	
Beneficial for work		32 (6)
Accessing education/training		9 (2)
Well-being		50 (9)
Online shopping		33 (6)
Information seeking	**95 (18)**	
Keeping up to date with news		32 (6)
General information searching		63 (12)
Technology	**36 (7)**	
Developing digital skills		21 (4)
Specific digital platforms (e.g. Zoom)		15 (3)

### Social connections

In relation to social connections, Table 4 shows that keeping in touch with friends, family and work colleagues was identified as a best thing about the Internet during the pandemic by over half of participants (52%):

Keeping in touch with my friends. It has given me comfort seeing them know that they’re ok.

It’s been nice to keep in touch with people like my mum and dad and my brother.

Also related to social connections, the second most common best thing was taking part in live online activities of groups (identified as a best thing by 16%):

Really busy schedule of online activities. The drama, afternoon tea, craft events, science fiction events.

I go to a club online and they play bingo on Tuesday, that’s been my favourite thing to do.

### Entertainment

Activities relating to entertainment were identified by 31% of participants as a best thing about the Internet during the pandemic. These activities included watching videos online (e.g. YouTube) and individual activities such as listening to music and online puzzle games.

Watching stuff on YouTube, Netflix and BBC iPlayer. I love it.

I have really liked the Christian radio which has helped me to focus on my faith.

### Life, work and education

Activities connected to life, work and education were identified by 23% of participants. These responses included specific well-being benefits; benefits to using the Internet for work; online shopping and access to education or training.

It’s helped keep me sane.

Having something to keep me occupied mostly it’s the only reason I am able to function most of the time -other than my partner. My partner is my first lifeline, the second is the internet and the third is my cat!

Being able to work from home while being safe.

You do not have to wait in a line when you shop online.

### Information seeking

Information seeking activities were identified by 18% of participants. This theme included general information searching (e.g. weather, ideas for travelling) as well as searching for general news and news relating to COVID-19:

Being able to keep up to date with events. News channels do a good job but internet handy for local news. Pop in postcode and find out rules that are relevant to you.

Doing research for things like animals and space.

### Technology

Just 7% of participants identified technology (the benefits of specific platforms and developing digital skills) as the most important benefit to Internet use during the pandemic:

I have learnt to do more because of zoom, the share screen has been really helpful for me in filling in forms.

Participants were asked, ‘During the coronavirus situation, what have been the worst things about having the Internet?’ Table 5 shows the number and percentage of participants mentioning each ‘worst thing’.

**Table 5.** Coded worst things about the Internet during the coronavirus pandemic.

	Number (and %) of participants mentioning theme (total sample = 485)	Number (and %) of participants mentioning item (total sample = 485)
Nothing	**192 (40)**	192 (40)
Technology	**137 (28)**	
Internet connection difficulties		114 (24)
Access barriers (cost, payments, accessibility)		11 (2)
Lack of digital skills		12 (2)
Online Harm	**120 (25)**	
Bullying, unwanted contact, harassment		43 (9)
Cybercrime (scams, hacking, fraud)		36 (7)
Upsetting content		26 (5)
Fake news		15 (3)
Threats to well-being	**70 (14)**	
Being online too long and inactivity		26 (5)
Restricts in-person contact		24 (5)
Overwhelming (too many people, anxiety)		9 (2)
Privacy concerns		5 (1)
Spending too much money online		3 (1)
Restrictions by others		2 (<1)
Clashing of online activities		1 (<1)

Of those who responded (*N* = 485), 192 (40%) responses were coded as ‘Nothing’. While 131 participants did not answer this question or responded with ‘don’t know’, a deliberate response of ‘nothing’ was coded separately. Participants who responded with ‘nothing’ often expanded their response to explain how important the Internet had been to them during the pandemic:

Nothing, I’ve just been on it all the time.

It’s all good, if I didn’t have the internet, I would be very bored and not able to talk to anyone or join in in meetings.

I haven’t got any bad opinions about the internet, it has all been good.

Across the range of responses that specifically identified a worst thing, codes were categorised into three themes. In order of number of mentions by participants, these were technology, online harm and threats to well-being.

### Technology

Worst things in relation to technology were mentioned by 28% of participants, with 24% identifying Internet connection difficulties as the worst thing about the Internet and others highlighting problems with access barriers and a lack of digital skills:

When it doesn’t work!

When it goes down, it affects my whole routine in terms of keeping in contact with others and my activities

the worst thing is that we see a lot of National Trust places that we want to go to but me and my wife have a lot of trouble booking them – it is like it is teasing us saying this is what you could be doing -but we can’t because the system is too difficult.

It can break -you can miss payments, it can be difficult to get back on and talk again.

I cannot use the computer without help.

### Online harm

In relation to online harm, participants identified bullying (9%), cybercrime (including hacking, scams and fraud) (7%), upsetting content (5%), and fake news (3%) as being worst things about the Internet:

I don’t like people arguing online . . . it annoys me so much that I feel sometimes like shutting down my computer.

I am worried about spam emails and people hacking into my account.

If you start looking about information on coronavirus that might not be true. You have to be careful of some places.

### Threats to well-being

Threats to well-being were identified by 14% of participants as a worst thing about the Internet during the pandemic. This theme included being online too long; restricting in-person contact with friends, family and colleagues; the Internet being overwhelming; concerns about privacy and oversharing of information; and spending too much money when online shopping:

Going on it too long and not going outside. I do go outside but not for too long.

Being glued to the internet all the time . . . it gives me a headache and I don’t want that.

I used to go to the self-advocacy meetings but when the meetings moved to zoom I had to stop going because I do not like so many people in one meeting.

Everybody knows your business.

I buy things I don’t really need.

In terms of maintaining interest in taking part in online activities, 56% of participants were keen to take part in online activities the whole way through the COVID-19 pandemic and 16% said that they were not keen at first but had become so at the time of the interview.

Participants were also asked if they wanted their lives to go back to how they were before the pandemic or if they wanted some things to be different to how they were before the pandemic. In total, 55 participants (9.2%) responded to this question specifically identifying they would like to keep something relating to Internet use. These responses identified issues such as benefits of working at home, increased opportunities for online friendships, opportunities to take part in activities in the evenings and opportunities for more people with intellectual disabilities and ‘high profile people’ to be able to attend campaigning meetings due to fewer barriers such as those associated with travelling:

I would like some things to be a bit different. In some ways it has been better because I have never been so busy. That is because it is now so much easier to connect to people online.

I have also been able to get to more work meetings than before with zoom. It would be good if this stays, and you can go to the meeting or join by zoom giving a choice and allowing more people to have a voice.

## Discussion

As far as we are aware, this dataset is the largest to date examining the digital experiences of adults with intellectual disabilities during the COVID-19 pandemic. Most participants (89.8%) had access to the Internet at home with 88.9% using it daily, which is comparable with the 89% of the general population in the United Kingdom in January to March 2020 (ONS, 2020b). Supporting previous research (e.g. Helsper and Reisdorf, 2017) findings from this study identified that younger adults (16–34) were the biggest users of the Internet, they were more likely to use the Internet, to spend more time online and to use the Internet for a range of activities. These findings support Martin et al.’s (2021) conclusion that older people in particular might require more support to join in with the digital world. However, age differences were not apparent for some activities; doing online activities with others, using the Internet for paid or voluntary work, and doing other activities alone. Participants most commonly used the Internet for being with family and friends online, social media or doing online activities with other people. In response to an open-ended question, participants most commonly identified social connections as being the best thing about the Internet during the pandemic. People who lived with family were the most likely and people who lived with other people with intellectual disabilities were the least likely to use social media. People who reported themselves to be never or hardly ever lonely were more likely to use the Internet for online activities with others and play video games with others online supporting suggestions from previous research that active (rather than passive) Internet use can be associated with reduced levels of loneliness (Yang, 2016). Although 40% of participants said that nothing was bad about Internet use during the pandemic, issues with technology (especially Internet connection), concerns about online harm and threats to well-being were identified as worst things about using the Internet.

There is a little consensus among researchers about the effects of Internet use on loneliness, with the content people are exposed to, the intensity of online interactions and consideration for what time spend online is replacing offline, not always being considered (Kaye, 2022). However, there is some evidence that Internet use for disabled people can sometimes be associated with reduced loneliness (Duplaga and Szulc, 2019). In England, the evaluation of the Digital Lifeline initiative found that following being given devices and support to use them, the majority of people with intellectual disabilities felt less lonely and more connected (Good Things Foundation, 2021). In this research, participants who took part in online activities with others or played video games with others online were more likely to report themselves as less lonely. In total, 9% of participants specifically mentioned something relating to well-being as the most important benefit to Internet use during the pandemic. The potential for supporting wellbeing is important when considered with the emerging evidence that for people with intellectual disabilities, lockdown may have had a harmful effect on emotional well-being (Amor et al., 2021; Lake et al., 2021).

While Araten-Bergman and Shpigelman (2021) found that video communication was not perceived as effective in filling the gap created by face-to-face contact, 72% of participants in this study were keen to take part in online activities at the time of interview (responded that they were ‘keen all the way through’ or ‘were not keen at first but am now’). The findings suggest that many people with intellectual disabilities have valued Internet use, in particular, in relation to social connections. Social activities (being with family and friends online, social media or doing online activities with other people) were the most common online activities and participants identified social activities being the best thing about the Internet. While these findings are similar to other research that has highlighted the importance of online social connections for the wider population during the pandemic (e.g. Saud et al., 2020), these findings are particularly important for people with intellectual disabilities in a context where many families reported substituting in-person visits with their relatives with remote communication (Araten-Bergman and Shpigelman, 2021; McCausland et al., 2021) and where technology played an essential role in accessing services, support and a way to connect with peers (Lake et al., 2021; Navas et al., 2021). A commonly identified worry during the pandemic by people with intellectual disabilities was social isolation (Flynn et al., 2021b), so it is perhaps unsurprising that people with intellectual disabilities identified social connections as the best thing about the Internet during the pandemic. As well as social connections and the ease of use of technology, people with intellectual disabilities also highlighted entertainment, life, work and education benefits, and information seeking as being good things about the Internet during the pandemic. These themes, as well as closely aligning with the United Nations Convention on the Rights of Persons with Disabilities, align with ways that the wider population engaged with the Internet during the pandemic (Pew Research Center, 2021b).

Previous research has often highlighted barriers to accessing online worlds relating to the impact of gatekeeping or safeguarding (Barlott et al., 2019; Chadwick, 2019; Heitplatz et al., 2021). Staff in residential homes might perceive social media as a social risk (Ramsten et al., 2019) and social work students perceive social media to be riskier and less safe for people with intellectual disabilities (Chiner et al., 2020). In this study, in terms of living situation, people living with other people with intellectual disabilities were less likely than people living with family to have access to the Internet at home, they were least likely to use the Internet for social media and less likely to spend more than 4 hours a day on the Internet. Previous research has shown that there is often a mirroring of offline and online lives and that people socialise online with offline friends (Livingstone, 2008; Valentine and Holloway, 2002). People with intellectual disabilities living with other people with intellectual disabilities may have more restricted lives offline (Emerson and McVilly, 2004) meaning their online worlds would be similarly restricted making social media less appealing (Raghavendra et al., 2018).

Many people with intellectual disabilities identify their disability as being the biggest barrier to inclusion (Good Things Foundation, 2021). While this study has identified perceived benefits to Internet use during the pandemic, there are important concerns that persist in causing barriers to accessibility for people with intellectual disabilities. The worst thing about the Internet that was most commonly mentioned was problems with Internet connection. For participation in online activities to be truly accessible to people with intellectual disabilities, unreliable Internet connections can be problematic as solving these problems are often not easy to navigate. Of participants who identified a worst thing about the Internet during the pandemic, 25% identified online harm, including scams, hacking and fraud. While some of these participants were concerned about the threat of harm rather than having experienced harm, findings from the first wave of the research found that 20% of participants (Flynn et al., 2021a) reported that someone has tried to scam or cheat them out of money over the previous 4 weeks.

## Limitations

This research was part of a larger study that took place at three timepoints and explored a wide range of experiences of people with intellectual disabilities during the COVID-19 pandemic in the United Kingdom. The study was responsive to urgent and important issues for people with intellectual disabilities and the people who support them. As such, there was limited scope to explore some issues relating to Internet use in more depth (e.g. it was not possible to track change over time nor was it feasible to explore detail about specific devices and how actively engaged people were in online activity).

Research that has taken place during the COVID-19 pandemic has suggested that barriers to digital inclusion for people with intellectual disabilities persist (Chadwick et al., 2022). It is therefore important to highlight that this study may have included participants who by the time of data collection in 2021, were particularly digitally skilled. Recruitment to the study was often through digital connections and data collection methods included interviews by whatever mode the participant preferred, with 60% of people choosing Zoom. Just 4% of participants identified learning new digital skills as the ‘best thing’ about the Internet during the pandemic, a figure that is perhaps surprisingly low. Responses to open-ended questions suggest that the simplicity of using platforms such as Zoom meant that participants who were new or inexperienced users might not have associated this with the development of their own digital skills (e.g. ‘Zoom has been great and easy to use’; ‘using FaceTime and Zoom, benefit even if not tech savvy’).

## Conclusion

Previous research has often highlighted risks and barriers to accessing online worlds for people with intellectual disabilities but findings from this research suggest that participants were having a largely positive experience of Internet use during the COVID-19 pandemic. In line with the experiences of non-disabled people during the COVID-19 pandemic, this research has identified that the most commonly identified uses and benefits were social. It should be a priority to support ongoing opportunities for maintaining social connections and expanding opportunities for online friendships. As a facilitator to digital inclusion, living with family meant that people with intellectual disabilities were more likely to have access to the Internet at home and more likely to use social media than people in other living situations. Many participants did not perceive there to be a worst thing about the Internet, but for those that did, connection difficulties were of primary concern. It is important to identify these concerns because barriers that are faced by the wider population can be more problematic for people with intellectual disabilities and prevent digital inclusion.
